# Draft genome sequence of *Bacillus cereus* HS120, a probiotic candidate isolated from the gut of hilsa (*Tenualosa ilisha*)

**DOI:** 10.1128/mra.00151-26

**Published:** 2026-04-30

**Authors:** Farzana Yeasmin, Md. Rony Babu, Promi Saha, Santu Biswas, Arpita Guha, Tofazzal Islam, Dipali Rani Gupta, Md. Mahbubur Rahman

**Affiliations:** 1 Institute of Biotechnology and Genetic Engineering, Gazipur Agricultural University 198780, Gazipur, Bangladesh; Montana State University, Bozeman, Montana, USA

**Keywords:** *Bacillus cereus*, hilsa (*Tenualosa ilisha*), gut microbiome, probiotic, antimicrobial peptides, biosynthetic gene clusters

## Abstract

*Bacillus cereus* HS120 was isolated from the stomach of a healthy hilsa (*Tenualosa ilisha*) in Nabogonga River, Bangladesh. Here, we present a 5,673,590-bp draft genome with 35.23 % GC content, which encodes numerous biosynthetic gene clusters for secondary metabolites and lacks detectable virulence factors, supporting its potential as an aquaculture probiotic.

## ANNOUNCEMENT

**Fig 1:**
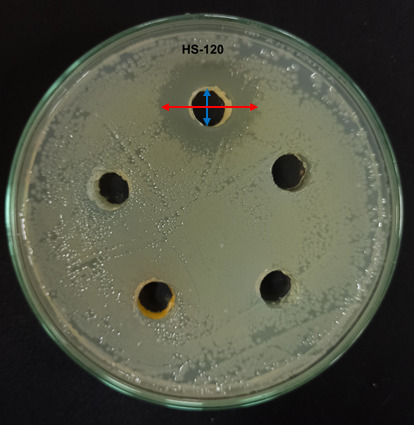
Inhibitory effects of cell-free culture filtrate of *B. cereus* HS120. Antimicrobial activity was evaluated using the agar well diffusion method. Fish pathogenic *Aeromonas veronii* B55 was uniformly spread onto Mueller-Hinton agar plate. Wells (blue arrow) were aseptically punched, and 50 µL of cell-free culture filtrate of HS120 was added to one well. After 48 h of incubation, a clear zone (red arrow) surrounding the well indicated inhibition of the pathogen.

Hilsa (*Tenualosa ilisha*), the national fish of Bangladesh, contributes approximately 12 % of the country’s total fish production (1). The anadromous hilsa exhibits notable disease resilience, suggesting that its associated microbiome plays a key role in host health and pathogen defense. Recent metagenomics studies reveal the probiotic potential of the hilsa microbiome (2, 3). To explore hilsa-associated probiotic bacteria, *Bacillus cereus* HS120 was isolated from the stomach of a healthy hilsa from the Naboganga River (23.26° N, 89.24° E), Bangladesh. Stomach tissue was aseptically processed, serially diluted, plated on Zobell agar, and incubated at 28°C for 24 h. A single colony was purified by repeated streaking. Cell-free culture filtrate of HS120 exhibited potent antagonistic activity against the fish pathogens *Aeromonas veronii*, *Streptococcus agalactiae*, and *Enterococcus faecalis* (Fig. 1 ) in an *in vitro* assay (4).

Genomic DNA was extracted from a 24-h culture grown in Zobell broth at 28°C with shaking at 150 rpm under aerobic conditions using the GeneJET Genomic DNA Purification Kit (Thermo Fisher Scientific, USA). DNA quality and quantity were assessed using a Qubit 4.0 fluorometer and a NanoDrop 2000 spectrophotometer, respectively. High molecular weight genomic DNA was used without intentional shearing to preserve long-read sequencing capability, and no explicit size selection step was performed. A sequencing library was prepared using the Oxford Nanopore Ligation Sequencing Kit (SQK-LSK109/114), purified with AMPure XP beads, and sequenced on a MinION Mk1B device (5). Base-called was performed using Guppy (Oxford Nanopore Technologies) with high-accuracy model.

A total of 67,954 generated raw reads were assessed using NanoPlot v1.46.1 (6), adapter trimmed with Porechop v0.2.4 (7), and filtered (Q > 10; length > 1,000 bp) with Filtlong v0.3.1 (8). *De novo* assembly was performed with Flye v2.9.6 (9, 10) and polished with Medaka v2.1.1 (11). Assembly quality was evaluated using QUAST v5.3.0, and annotation was performed using the NCBI Prokaryotic Genome Annotation Pipeline (12). Additional genomic features were studied using RAST Server v2.0 (13), VirulenceFinder (14), and PlasmidFinder v2.0.1 (15). All analyses were performed using default parameters.

The draft genome of *B. cereus* HS120 comprises 5,673,590 bp (GC content 35.23%), assembled into six contigs (largest contig: 5,331,035 bp; smallest contig: 5,049 bp) with approximately 94 × coverage. The N50 value was 5,331,035 bp. A total of 5,843 genes were predicted, including 5,510 protein-coding sequences and 156 RNA genes. Average Nucleotide Identity analysis (OrthoANIu) ( 16) revealed 98.80% similarity to *B. cereus* (BioSample ID: SAMN44839476), confirming species identity. Genomic mining identified 14 biosynthetic gene clusters via antiSMASH 8.0.4, including NRPS, RiPP, and betalactones (facilitating niche adaptation) ( 17), and two antimicrobial peptide loci via BAGEL4 (18). Notably, no plasmids, antibiotic resistance genes (via ResFinder 4.7.2), or virulence-associated genes (VFDB) were detected (15, 19, 20). Functional analysis highlighted genes for nutrient assimilation (metallocarboxypeptidases and aminopeptidases) ( 21, 22), sporulation (supporting environmental persistence) ( 23), TasA-mediated biofilm formation (facilitating mucosal attachment) ( 24), and iron-scavenging systems (bacillibactin and petrobactin) ( 25). This is the first genome report of a *B. cereus* strain from hilsa gut exhibiting probiotic-associated traits, supporting its safe application as a probiotic candidate for sustainable aquaculture.

## Data Availability

The complete genome sequence of *Bacillus cereus* HS120 has been deposited in GenBank under the accession number JBTXPM000000000. The raw sequencing reads have been deposited in the NCBI Sequence Read Archive (SRA) under the accession number SRR37258482. The data can be found under BioSample accession number SAMN53301936 and BioProject accession number PRJNA1366063.
